# Phosphorus, Combined with Malaria, the Cause of Yellow Fever

**Published:** 1879-02-20

**Authors:** Thos. W. Hewitt


### *PHOSPHORUS, COMBINED WITH MALARIA, THE CAUSE OF YELLOW FEVER.*

BY THOS. W. HEWITT, M.D.

Phosphorus is well known to exist in the sea of Southern zones, is dashed upon decks of ships, and can be seen there at night, and consists of myriads of animalcules carried by it. It exists among the isles of the Antilles, from whence yellow fever is said to come. Phosphorus exists on logs undergoing decay in the woods of the Mississippi Valley, and can be seen almost any night on the new grounds here in Arkansas called deadenings. It is very abundant. It is seen in the swamps where the water has gradually left the surface, thus exposing it by night and is familiarly known here as fox-fire. In the cesspools of cities and towns it exists, and has been manufactured from them. We know what malaria is. We say malaria poisons in degrees, giving grades of effect; but what is the poison? Animalcules conveying the poison? Yes; but what is it? I say, for yellow fever, it is phosporus. Can animalcules carry it? Yes; the lightning-bug carries it. Myriads of lesser insects carry it in the sea, and lesser still, the malaria animalcule carries it.

Further, what is a case of yelloy fever? It is poisoning. Take a case of poisoning by phosphorus. We have depression of the heart's action, a high internal temperature, a slow pulse; the tongue and mouth are identical with yellow fever; the stomach is exceedingly irritable (gastrititis it is called); jaundice follows; the urine is similar; the black vomit has been seen in the disease. Has any treatment reached it save an antidotal one? I have acted upon it, and have had it to succeed with startling success in the few cases I have had here and in this vicinity.

I have but very badly brought to my memory the above without any of my notes at hand. Sir Wm. Henry Day, of London, England, is my authority on phosphorus poisoning, and Flint's latest edition of Practice of Medicine on Gastrititis. I hope you will refer to his Hæmaturia as well. I am anxious for you to investigate this cause, with a view to further proof, and do not deny it without proof.—*Louisville Med. News.*

---

**Permanganate of Potash.**—Permanganate of potash is a valuable local remedy in the treatment of erysipelas. Two grains of it dissolved in an ounce of water, and applied as a wash, will soon subdue the inflammation. Tincture of iron should be given every three hours, until a cure is effected. Tincture of veratrum is the best remedy to subdue the fever, as it also subdues the inflammation.—*Ibid.*

